# Formation of Nanocones on Highly Oriented Pyrolytic Graphite by Oxygen Plasma

**DOI:** 10.3390/ma7032014

**Published:** 2014-03-11

**Authors:** Alenka Vesel, Kristina Eleršič, Martina Modic, Ita Junkar, Miran Mozetič

**Affiliations:** Plasma Laboratory, Institute Jozef Stefan, Jamova 39, Ljubljana 1000, Slovenia; E-Mails: kristina.elersic@ijs.si (K.E.); martina.modic@ijs.si (M.M.); ita.junkar@ijs.si (I.J.); miran.mozetic@guest.arnes.si (M.M.)

**Keywords:** highly oriented pyrolytic graphite (HOPG), oxygen plasma treatment, nanocone formation, platelet adhesion

## Abstract

Improvement in hemocompatibility of highly oriented pyrolytic graphite (HOPG) by formation of nanostructured surface by oxygen plasma treatment is reported. We have showed that by appropriate fine tuning of plasma and discharge parameters we are able to create nanostructured surface which is densely covered with nanocones. The size of the nanocones strongly depended on treatment time. The optimal results in terms of material hemocompatibility were obtained after treatment with oxygen plasma for 15 s, when both the nanotopography and wettability were the most favorable, since marked reduction in adhesion and activation of platelets was observed on this surface. At prolonged treatment times, the rich surface topography was lost and thus also its antithrombogenic properties. Chemical composition of the surface was always more or less the same, regardless of its morphology and height of the nanocones. Namely, on all plasma treated samples, only a few atomic percent of oxygen was found, meaning that plasma caused mostly etching, leading to changes in the surface morphology. This indicates that the main preventing mechanism against platelets adhesion was the right surface morphology.

## 1. Introduction

Thrombogenic response of the body is very complex and quite poorly understood, especially in connection with artificial surfaces which are in constant contact with blood (e.g., vascular grafts, artificial heart valves, *etc*.). In order to improve hemocompatibility of blood connecting materials it is important to understand the mechanisms of blood interaction with surface. It is now generally accepted that the blood plasma proteins are the first to come in contact with the artificial surface and adsorb on the surface of biomaterial. Adsorbed proteins are then recognized by platelet receptors which mediate platelet adhesion on the surface [1]. This promotes platelet activation, aggregation and subsequently thrombus formation. Traditional methods for improving blood compatibility of implants were focused on the minimization of blood interaction with materials; however, it is nowadays believed that the biomaterial surface should interact with the biological material to elicit the appropriate biological response [2]. Treatment of materials with chemicals or coating materials with bioactive coatings or even drug eluting coatings are common approaches in the direction of hemocompatible devices [3]. However, body responses can overcome theoretical predictions. In spite of many efforts to fabricate the biomaterial that would possess ideal mechanical and hemocompatible properties, there are still no such materials produced on the biomedical market.

Mechanical heart valves need to be designed to fit the particular blood flow through the spaces of the heart (pressure differences), with emphasis on producing more central flow and reducing blood clots [4,5]. They also need to have good anticoagulation properties to control thromboembolisms [6,7]. Mechanical heart valves can be fabricated from different biomaterials like alumina [8,9], titanium [10,11], polyester [12–14], polyurethane [15,16] and carbon-based materials like pyrolytic carbon [17,18], graphite [19,20] and diamond-like carbon (DLC) [21,22]. Indeed 95% of all mechanical heart valve prostheses are made completely, or at least partially from pyrolytic carbon [23]. Often graphite in combination with pyrolytic carbon is used [24]. Therefore, pyrolytic carbon is regarded to be one of the leading materials in mechanical heart valves replacements [25]. This material is also suitable for sterilization since it can withstand autoclaving and is not degraded in physiological environments. Also, its chemical inertness is more excellent compared with many other materials suitable for application as heart valves. However, the hemocompatibility of valves made from pyrolytic carbon are still not adequate enough. Therefore, new approaches based on polymeric materials coated with antithrombogenic agents such as heparin and fucoidan, have been proposed to improve the hemocompatibility of medical devices [3,26,27]. Although no material has been found truly biocompatible, many cardiovascular devices function with low or acceptable risks of complications [28].

One possible way to improve the hemocompatibility of materials is treatment with gaseous plasma [29–31]. In our recent paper, we have shown that we can obtain antithrombogenic polymer surface only by treatment in oxygen plasma [32], without need for further grafting of plasma-treated surface with specific molecules or binding of heparin which is commonly applied method. It is well known that polymeric materials are easily functionalized upon treatment with gaseous plasma [33,34], however, this is not the case for graphite materials [35,36], where concentration of carbon-oxygen functional groups is much smaller compared to plasma-treated polymer. Graphite is less easily functionalized due to its specific crystalline structure and the wettability of the surface upon treatment with oxygen plasma is not optimal. The shortage of functional groups and low wettability of the surface may be overcome by modification of the surface morphology [37,38]. It is known that the wettability of many materials depends not only on the surface functional groups but also on its roughness, which can also have a significant influence on the biological response. Numerous studies have dealt with the cell behavior on surfaces with different roughness as cells can sense the chemistry and topography of the surface to which they adhere [39–43]. It has been shown that nanometric features can elicit specific cell response [43,44]. Thus, by properly adjusting surface topography on a micro and nanometer scale it is possible to obtain a specific biological response from cells.

Uniform modification of the surface topography leading to formation of nanostructures on an otherwise perfectly flat material can be achieved by treatment with oxygen plasma, using appropriate parameters. In the present paper we are presenting surface properties and hemocompatibility response of nanostructured surface of highly oriented pyrolytic graphite (HOPG) formed after treatment with highly non-equilibrium oxygen plasma. Chemical structure of HOPG is similar as for pyrolytic carbon but has much higher orientation [45]. Therefore, it can be a good alternative for use as a material for mechanical heart valves.

## 2. Results and Discussion

### 2.1. Surface Characteristics of Plasma Treated HOPG

HOPG samples were treated in oxygen plasma for different treatment times and then the surface was analyzed by scanning electron microscopy (SEM), water contact angle measurements (WCA), atomic force microscopy (AFM), X-ray photoelectron spectroscopy (XPS) and tested for hemocompatibility (HEMO). For easier reading of the paper, the sample notation with short description and a list of applied experimental methods is shown in Table 1. More details can be found in experimental part at the end.

**Figure 1.**
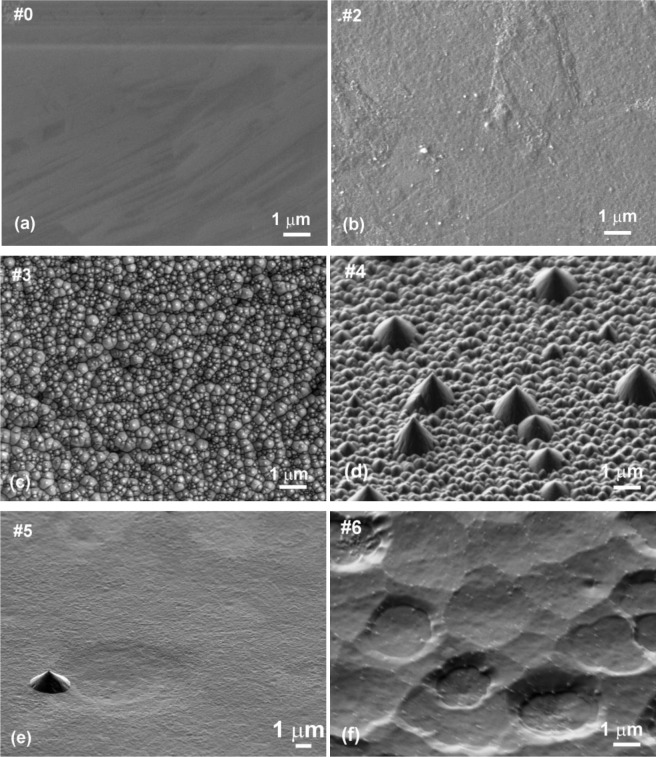
SEM images of untreated and plasma treated highly oriented pyrolytic graphite for (**a**) untreated; (**b**) 5 s of treatment; (**c**) 15 s of treatment; (**d**) 20 s of treatment; (**e**) 30 s of treatment and **(f)** 40 s of treatment. Radiofrequency (RF) plasma created at oxygen pressure of 30 Pa, flow rate of 22 L/s and nominal power of 700 W was used for sample treatment.

**Figure 2.**
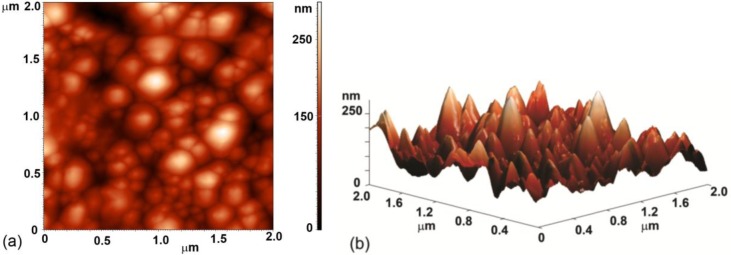
(**a**) 2D and (**b**) 3D Atomic force microscopy (AFM) image of highly oriented pyrolytic graphite (HOPG) sample (#3) after 15 s of plasma treatment.

Evolution of surface morphology of plasma treated HOPG samples with treatment time as determined by SEM is shown in Figure 1. No effect on the surface morphology was observed in the first ten seconds of treatment (samples #0–#2). However, interesting morphological changes were observed after 15 s of treatment as the surface was fully covered with small nanocones, as seen in Figure 1c (sample #3 from Table 1). The height and width of the nanocones measured on the surface of this sample with AFM was about 100 nm and 150 nm, respectively (Figure 2). Longer treatment times ~20 s (sample #4) resulted in appearance of individual large cones with the size of about one micron (Figure 1d). Between these large cones we can still observe surface densely covered with small nanocones. This was no longer the case after 30 s of treatment (sample #5), where only some large cones remained, while small nanocones between them have disappeared (Figure 1e). The surface between large cones is now quite smooth and similar to the untreated sample. At very long treatment times (more than 40 s, sample #6) also large cones disappeared and shallow pits are observed (Figure 1f). From Figure 1 we can thus conclude that nanostructures on the surface as shown in Figure 1c,d are observed only for a limited range of plasma parameters.

Such a rich surface nanomorphology as shown in Figure 1 probably resulted from the interaction of highly energetic oxygen particles with carbon atoms on the HOPG surface, which caused extensive nonuniform etching of HOPG. This in turn enabled creation of nanocones on the surface of originally perfectly flat material. The etching rate was determined by measuring the mass of a sample before and just after plasma treatment and was estimated to around 300 nm/s. Reactive particles created in the oxygen plasma interact chemically with the surface carbon atoms causing changes in surface topography due to pronounced etching. Such a behavior is explained by two competing mechanisms. The first one is selective etching of HOPG by interaction with energetic oxygen particles. This mechanism favors appearance of nanocones of sub-micron diameter and height. The other mechanism is thermal oxidation of HOPG, and this mechanism favors destruction of any sharp objects on the surface of a sample exposed to plasma. At low temperatures, the first mechanism prevails and this is why nanocones are observed on the samples treated with plasma up to about 15 s. The second mechanism becomes predominant after longer treatment time due to increase in the sample temperature during the treatment.

**Figure 3.**
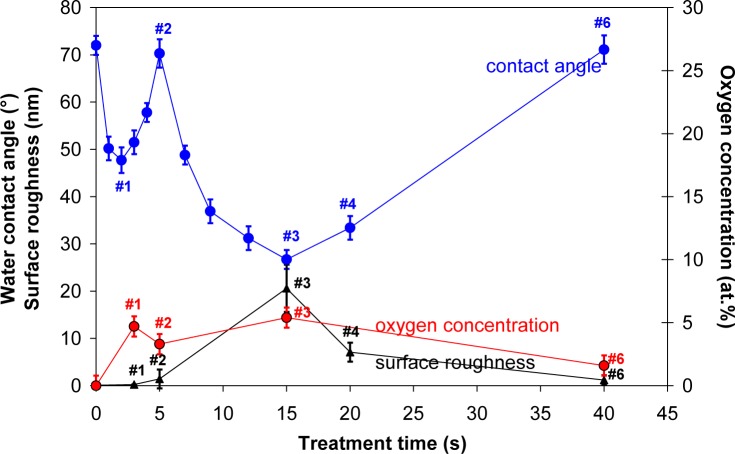
Water contact angle, surface oxygen concentration and surface roughness *versus* plasma treatment time.

Contact angle of a water drop on plasma treated HOPG samples is shown in Figure 3. First, a small minimum is observed at 3 s of treatment, where contact angle dropped from initial 72° to about 48° (sample #1). This minimum is then followed by a maximum, where contact angle increased to about 70° (sample #2). With further treatment time second minimum is observed, which is very deep and it appeared at 15 s of plasma treatment (sample #3). Here, the contact angle was only 25°. This second deep minimum corresponds to the case where surface was fully covered with nanocones (Figure 1c), while in the case of the first minimum, the surface was still smooth (Figure 1b) and quite similar to the untreated one. So therefore, the first minimum can be explained only by surface removal of carbon contaminants from the surface, as will be shown later in the text, while the second minimum can be explained by the formation of nanorough surface. This hypothesis is further supported by AFM measurements of the surface roughness which is also shown in Figure 3. We can see that the minimum contact angle is obtained at the same treatment time as the maximum surface roughness. At very long treatment times the contact angle increased again and at 40 s (sample #6) of treatment practically reached the initial value. This is due to the fact that nanostructures have disappeared at longer treatment times as described before.

**Figure 4.**
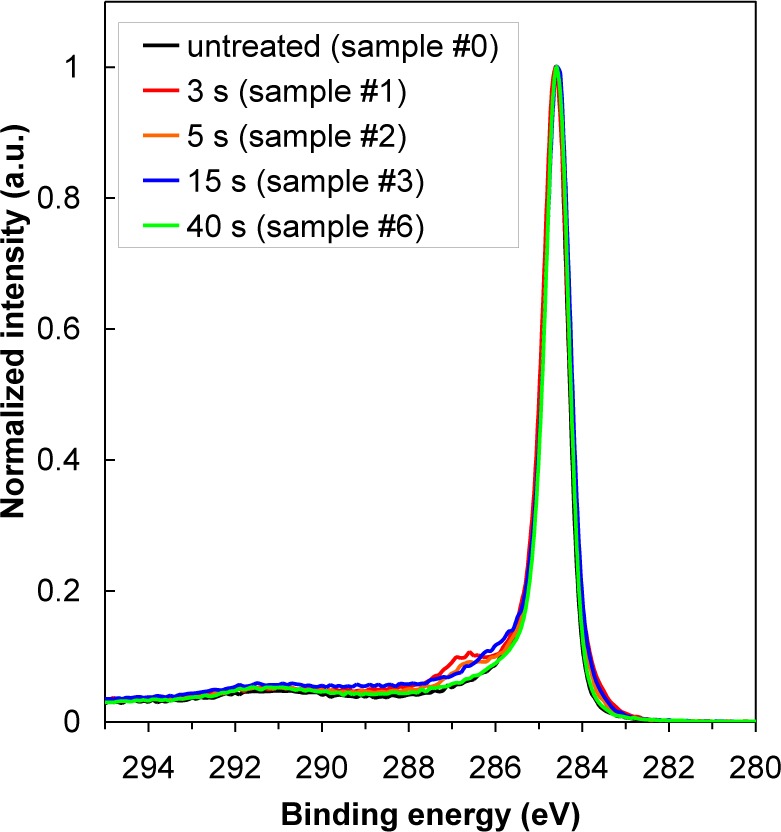
High resolution XPS spectra of C1s for plasma treated HOPG samples.

In Table 2 is shown oxygen concentration as determined by XPS. For better comparison, the results from Table 2 are plotted in Figure 3 as well. The highest oxygen concentration on the HOPG surface was found at 15 s of treatment (sample #3), while oxygen concentration at 40 s of treatment has significantly decreased (sample #6). Indeed, variation in oxygen concentration between the samples is very low. Therefore, dramatic changes of water contact angles are more probably due to changes in the surface morphology (*i.e.*, formation of cone-like nanostructures) than due to surface functionalization with oxygen (Figure 4), since amount of oxygen on the surface is quite small (Table 2), too small to cause such significant changes in the surface wettability. As shown in Figure 4, there is only a slight difference between XPS spectra of carbon C1s for untreated and plasma treated HOPG samples. Concentration of C–O bonds that are formed during surface oxidation by plasma is thus very small. Furthermore, we can also observe that C–O peak in Figure 4 is the most intense at 3 s of treatment (sample #1), while later its intensity is much lower. This can be explained by the removal of carbon contamination in plasma.

**Figure 5.**
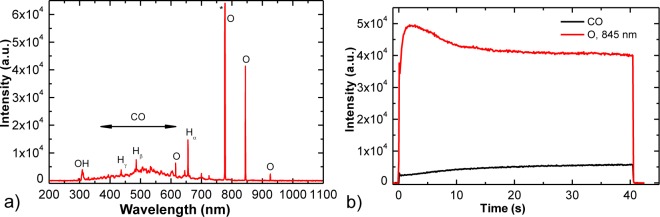
(**a**) Optical emission spectra (OES) spectrum of oxygen plasma during treatment of HOPG sample and (**b**) time evolution of CO peak intensity.

XPS results also indicate that oxygen radicals from plasma attack carbon atoms at surface via C–O bond formation and C–C bond breakage and leading to release of CO or CO_2_ molecules [46,47]. Oxidation of carbon probably takes place at the crystallographic borders which are weak points at HOPG surface. Formation of CO molecules was observed in OES spectra shown in Figure 5. The optical spectra revealed strong atomic oxygen lines (at 777 and 844 nm) and CO bands from 3rd positive as well as Angstrom transitions [48]. Furthermore, a broad continuum between 400 and 700 nm is observed, which was explained by partial overlapping of radiative transitions within CO molecule [48]. The results showed continuous decrease of oxygen lines and simultaneous increase of a CO line due to intensive oxidation of the graphite sample (Figure 5b). OH band and hydrogen atomic lines which are also observed in Figure 5a are due to desorption of water molecules from the surface of plasma reactor.

### 2.2. Hemocompatibility Response of Plasma Treated HOPG

**Figure 6.**
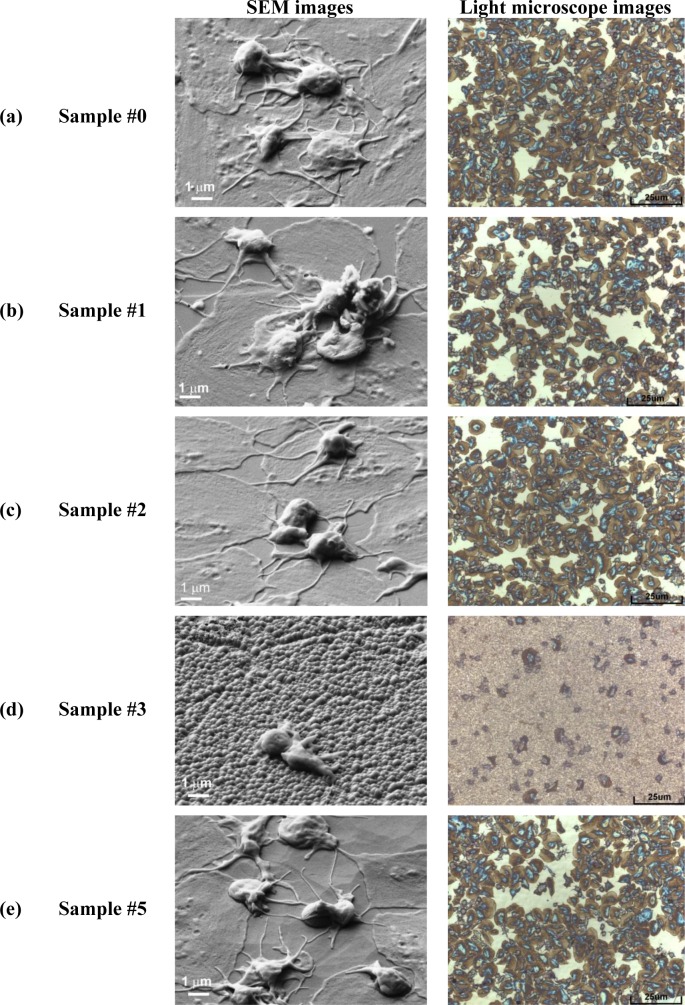
(**left**) SEM images of HOPG samples and (**right**) images taken by light microscope: (**a**) untreated sample; (**b**) plasma treated for 3 s; (**c**) plasma treated for 5 s; (**d**) plasma treated for 15 s with nanocones and (**e**) plasma treated at longer times—30 s.

**Figure 7.**
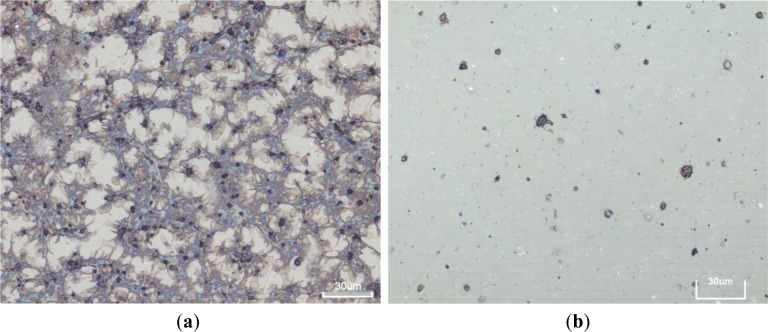
Images taken by light microscope of PET samples which were used as control samples: (**a**) untreated and (**b**) plasma treated PET. As expected, untreated PET surface is very thrombogenic and fully covered with platelets, while plasma treated PET surface has antithrombogenic effect [23].

After plasma treatment, the HOPG samples with different surface topography were tested to see their antithrombogenic properties. As a control material we have used polymer PET (polyethylene terephthalate). For the in vitro response the samples were incubated in whole blood and then platelet adhesion was determined from images taken by SEM and light microscopy. The results for HOPG samples are summarized in Figure 6 while for control samples are shown in Figure 7.

In Figure 6a is shown platelet adhesion on untreated sample (#0). In Figure 6b,c is shown platelet adhesion for plasma treated samples (#1, #2) before nanocones formation while in Figure 6d is shown the (#3) which is fully covered with dense nanocones (15 s of plasma treatment). Furthermore, in Figure 6e is shown the case for long treatment times (30 s of plasma treatment, sample #5), where small nanocones have already disappeared. A big difference in the number of adhered platelets between the surfaces covered with nanocones and those that are perfectly flat or have only large cones are observed from right side of the Figure 6. While the untreated surface (#0) as well as surfaces treated in oxygen plasma for short times (#1, #2) are fully covered by platelets (Figure 6a–c), only few of them are observed on plasma treated surface (#3) which was covered with small nanocones (Figure 6d). It seems that such uniformly and densely distributed nanocones on HOPG materials as seen in Figure 1c have very high impact on platelet adhesion shown in Figure 6d, since at longer treatment times (sample #5 in Figure 6e), when small nanocones disappear (Figure 1e,f), the situation regarding platelet adhesion is again similar to the case of untreated sample. Large micron-size cones as observed in Figure 1e for sample #5 were thus shown to have no influence on the platelet adhesion. Difference in adhesion of platelets on the surface treated for 15 s (sample #3, Figure 6d) can thus be explained by the differences in surface morphology and appropriate size and distribution of the nanocones, which prevent platelet adhesion.

From Figure 6 we can see another important difference: plasma treatment does not only have an impact on the number of adhered platelets, but also on their morphology (*i.e.*, activation). Namely, after contact with the foreign material platelets interact and adhere on the surface by the series of different reactions which induce platelet activation. One of the ways to determine the degree of platelet activation is by observing transformation of their morphology. In general platelets are classified into five groups according to their morphology; round, dendritic, spread-dendritic, spread and fully spread where round shape is nonactivated and fully spread is totally activated [46]. It can be clearly seen that no drastic changes in platelet morphology can be observed for samples #0, #1, #2 and #5 shown in Figure 6a–c,e, while the morphology of platelets in Figure 6d (sample #3) is very different. In Figure 6a–c,e we can observe spread platelets with many extending pseudopodia, which are connected with high platelet activation and thus high risk of thrombus reactions. It is also interesting, that in the case of a sample with the large nanocones (sample #5, Figure 6e), platelets are spreading not only on the flat HOPG surface between the large nanocones, but they can adhere also on the surface of the large nanocones themselves. So this means that while platelets can adhere to the large nanocones with dimensions of about 1 μm which are found on sample #5, the spreading and activation of them on the nanocones of smaller dimensions (100 nm or less) as those shown in Figure 1c for sample #3 is not favorable (Figure 6e). Namely, figure 6e shows that platelets are mostly in round non-activated form as hardly any extend pseudopodia and or spread platelet shape is noticed, which is connected with low platelet activation and thus lower risk of thrombus reactions.

### 2.3. Discussion on Platelet Adhesion

Let us now explain the possible reasons for observed differences in the platelet adhesion. Normally changes in adhesion properties of plasma treated samples are related to surface functionalization, different surface morphology and wettability. In our case, the changes in wettability were observed after different treatment time, but it should be mentioned that the changes in wettability are mostly influenced by the changes in morphology of the surface. The minimum water contact angle was measured on the surfaces treated for 15 s (Figure 3), which correlates well with the formation of nanocone structures (Figure 1b). Longer treatment time however showed increase in water contact angle and it reached practically initial value after 40 s of treatment. Interestingly the chemical composition of the surface was not much altered during treatment (Table 2). Increase in oxygen content from about 2 at% to about 5 at% was noticed already after 5 s of treatment and it did not change much after 15 s of treatment (6 at%). Prolonged treatment (40 s) resulted in a bit lower oxygen content (3 at%). It should be emphasized that these differences are not significant and they could be in the range of the experimental error of XPS measurement or they can be even attributed to the influence of surface roughness on XPS measurements. Other chemical elements were not found on surfaces of HOPG after plasma treatment. These results thus confirm that wettability in our case is mostly influenced by the change in the surface morphology, as oxygen content on 5 s (sample #2) and 15 s (sample #3) treated surface was almost the same, while the wettability was quite different. Moreover, the biological response between these two surfaces was also quite different, as reduced adhesion was only noticed on the samples treated for 15 s (sample #3). This again confirms that platelet adhesion in this case is mainly influenced by the surface morphology. The surface as seen from a platelets perspective is made of dots of nanocone tops. The contact area between nanocone tops and platelets is not sufficient for their adhesion on nanocones. A distance between created nanocones (about 0.2 μm) is also too small for platelets to comfortably sit and adhere between them. Therefore, marked reduction in platelet adherence in this case was correlated to the lack of available surface for platelets adhesion.

## 3. Experimental Section

### 3.1. Plasma Treatment

**Figure 8.**
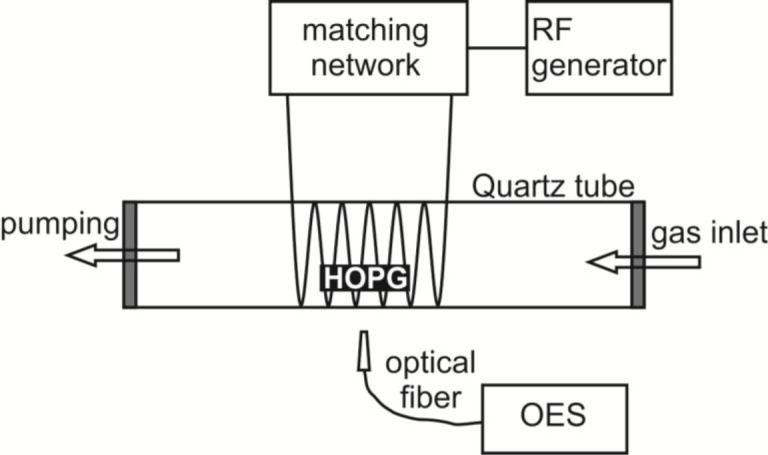
Scheme of the experimental plasma reactor.

Samples of HOPG (ZYH type from NT-MDT) were exposed to oxygen plasma created by inductively coupled electrodeless radio-frequency discharge in pure H-mode [49]. A scheme of such plasma reactor is presented in Figure 8. Plasma reactor was composed of a discharge tube made from Quartz glass. A length of the tube was 80 cm and a diameter was 4 cm. A coil of 6 wounds was mounted in the center of the tube. Rather large distance between the coil and the grounded flanges was applied in order to minimize the capacity coupling. Plasma was created by an RF generator coupled to the coil via a matching network. The generator operated at the standard frequency of 13.56 MHz. The nominal power was set to 700 W. The discharge chamber was pumped with a Roots pump with a nominal pumping speed of 400 m^3^ h^−1^ backed by a two-stage oil rotary pump with a pumping speed of 80 m^3^ h^−1^. Commercially available oxygen was added into the discharge chamber through a flow controller. The pressure was measured by an absolute vacuum gauge. The pressure was fixed to 30 Pa and the flow rate was 22 L/s. Such experimental conditions allowed for practically 100% dissociation of oxygen molecules in H-mode and substantial concentration of atoms and metastable excited states [49]. The HOPG samples were exposed to such extremely non-equilibrium oxygen plasma for different periods. During HOPG treatment optical emission spectra (OES) were recorded to follow plasma-surface interactions. OES spectra were recorded by using spectrometer AVASPEC 3648 (Avantes Ltd, Leatherhead-Surrey, UK) with the spectra resolution of 0.5 nm in the spectral range from 180 to 1100 nm. The integration time was set to 50 ms.

### 3.2. Surface Characterization

After plasma treatment the wettability was determined from water contact angles, and the concentration of oxygen on the surface was determined using X-ray photoelectron spectroscopy (XPS). Morphology of the HOPG samples was analyzed by Scanning electron microscopy (SEM) and Atomic force microscopy (AFM).

Chemical composition of the samples was analyzed by the XPS instrument TFA XPS (Physical Electronics Inc., Chanhassen, MN, USA). The base pressure in the XPS analysis chamber was about 6 × 10^−8^ Pa. The samples were excited with X-rays over a 400-μm spot area with monochromatic Al K_α1,2_ radiation at 1486.6 eV. The photoelectrons were detected with a hemispherical analyzer positioned at an angle of 45° with respect to the normal to the sample surface. The energy resolution was about 0.5 eV. Survey-scan spectra were made at a pass energy of 187.85 eV and 0.4 eV energy step. The high-resolution spectra were made at a pass energy of 23.5 eV and 0.1 eV energy step. The concentration of the elements on the sample surface was determined using MultiPak v8.1c software (Ulvac-Phi Inc., Kanagawa, Japan, 2006) from Physical Electronics, which was supplied with the spectrometer. XPS measurements were performed for two samples for each treatment condition.

AFM images of plasma treated HOPG were obtained with an atomic force microscope (AFM, Solver PRO, NT-MDT, Zelenograd, Russia) in the tapping mode in air at the room temperature. The surfaces were analyzed with standard Si cantilever with a force constant of 10 N/m and at resonance frequency of 170 kHz. The scanning rate was around 1.56 Hz. Surface roughness was measured on area 1 × 1 μm^2^.

SEM images of plasma treated HOPG samples were obtained by using field emission scanning electron microscope JSM-7600F (JEOL, Tokyo, Japan). SEM analyses were repeated two or three times for each treatment condition.

The surface wettability was measured immediately after plasma treatment by determination of the contact angle of a demineralized water drop in a static mode. A volume of a water droplet was 3 μL. A home-made apparatus equipped with a CCD camera and a PC computer was used for taking high resolution pictures of a water drop on the sample surface. Three samples were prepared for each treatment condition and for each sample five measurements were perform in order to minimize the statistical error. The contact angles were determined by software See System (Advex Instruments, Brno, Czech Republic) which enables fitting of the shape of a water drop and drawing the tangent to the water drop surface. The estimated error in contact angle determination was ±3°.

### 3.3. Hemocompatibility Testing

Hemocompatibility of plasma treated samples was tested by incubation of HOPG samples with blood. The amount of adhered platelets on the surface of plasma-treated HOPG samples was observed with light microscope Axio CSM 700 (Carl Zeiss Vision GmbH, Munich, Germany) and their morphology was analyzed by SEM.

Detailed preparation of the samples for light and SEM microscopy is as follows: The HOPG samples were incubated for 1 h with whole blood. Whole blood was drawn from healthy volunteers via vein puncture. The number of platelets in the blood was 1.1 × 10^9^ L^−1^. The blood was drawn into 9 mL tubes with tri sodium citrate anticoagulant (Sigma Aldrich Co., Taufkirchen, Germany), and the number of platelets in whole blood was counted (Cell-DYN 3200, Abbott Laboratories, North Chicago, IL, USA). Afterwards the fresh blood was incubated with HOPG surfaces in 24 well plates for 1 h at 37 °C with shaking at 300 RPM. Each sample (measuring 10 mm in diameter) was incubated with 1 mL of whole blood. After 1 h of incubation the samples were rinsed with PBS (phosphate-buffered saline) five times in order to remove blood residues. Adhered platelets were then fixed in 2.5% glutaraldehyde for 30 min at 4 °C, rinsed with distilled water and dehydrated through a series of graded ethanol solutions (50%, 60%, 70%, 80%, 90% and 96%).

Samples prepared for SEM analysis were further dried in a Critical Point Dryer and sputtered with a thin layer of gold. SEM images were taken by JEOL JSM-7600F (JEOL, Tokyo, Japan) microscope at accelerating voltage of 5 keV.

## 4. Conclusions

Oxygen plasma treatment caused a growth of densely distributed cone-like nanostructures on the surface of HOPG samples. Such cone-like nanostructures were only observed for a limited range of plasma parameters *i.e.*, after 15 s of oxygen plasma treatment. At prolonged treatment times, the rich surface topography was lost. On plasma treated samples only few atomic percent of oxygen was found meaning that plasma causes mostly etching, leading to changes in the surface morphology, which has resulted in formation of nanocones. Difference in the number of attached platelets between the surfaces covered with nanocones and those that are perfectly flat or have larger nanocones were found. The optimal results in terms of material hemocompatibility were obtained after treatment with oxygen plasma for 15 s, when both the nanotopography and wettability were the most favorable.

## Figures and Tables

**Table 1.** Sample notation and description.

Sample label	Treatment time (s)	Description	Experimental methods
#0	0	untreated	WCA, SEM, XPS, HEMO
#1	3	1st minimum in contact angle	WCA, XPS, HEMO
#2	5	maximum contact angle	WCA, SEM, XPS, HEMO
#3	15	2nd minimum in contact angle, nanocone formation	WCA, SEM, XPS, AFM, HEMO
#4	20	additional appearance of large nanocones	WCA, SEM
#5	30	only few large nanocones observed	SEM, HEMO
#6	40	all nanostructures disappeared	WCA, XPS

**Table 2.** Surface composition of plasma treated HOPG samples as determined by X-ray photoelectron spectroscopy (XPS).

Sample	Surface composition (at%)		
	C	O	O/C
0 s (sample #0)	99.4	0.6	0.01
3 s (sample #1)	95.3 ± 0.1	4.7 ± 0.1	0.05
5 s (sample #2)	96.7 ± 0.5	3.3 ± 0.5	0.03
15 s (sample #3)	94.6 ± 0.3	5.4 ± 0.3	0.06
40 s (sample #6)	98.4 ± 0.6	1.6 ± 0.6	0.02
